# Design and Evaluation of Self-Emulsifying Drug Delivery System (SEDDS) Of Carvedilol to Improve the Oral Absorption

**DOI:** 10.17795/jjnpp-16125

**Published:** 2014-06-21

**Authors:** Anayatollah Salimi, Behzad Sharif Makhmal Zadeh, Ali asghar Hemati, Sanaz Akbari Birgani

**Affiliations:** 1 Department of Pharmaceutics, Nanotechnology Research Center, Ahvaz Jundishapur University of Medical Sciences, Ahvaz, IR Iran; 2 Department of Pharmacology and Toxicology, Ahvaz Jundishapur University of Medical Sciences, Ahvaz, IR Iran

**Keywords:** Carvedilol, Self-Emulsifying Drug Delivery Systems, Oral Absorption

## Abstract

**Background:**

Self-emulsifying drug delivery system is an isotropic mixture of natural or synthetic oils, non-ionic surfactants or, one or more hydrophilic solvent and co-solvents/surfactant and polymer that improve bioavailability and increase solubility of poorly-soluble drugs.

**Objectives:**

This study was aimed to prepare and develop a stable formulation for self-emulsifying drug delivery system to enhance the solubility, release rate, and oral absorption of the poorly-soluble drug, carvedilol.

**Materials and Methods:**

The prepared self-emulsifying drug delivery system formulations were evaluated regarding their particle size, refractory index (RI), emulsifying efficiency, drug release, and rat intestine permeability.

**Results:**

The results showed oleic acid as oil with Labrafil as surfactant and Labrafac PG (propylene glycol dicaprylocapraye) as co-surfactant with hydroxypropyl methylcellulose and Poloxamer as polymer prepared stable emulsions with a refractive index higher than acidic medium and water. The particle size of formulations was influenced by the type of polymer so that the mean particle size in the self-emulsifying drug delivery system formulations containing hydroxypropyl methylcellulose have a higher particle size compared to Poloxamer formulations. The percentage of drug release after 24 hours (R24) for Poloxamer and hydroxypropyl methylcellulose formulations were 61.24-70.61% and to 74.26-91.11%, respectively. The correlation between percentages of drug released after 24 hours with type of polymer was significant. In permeation studies, a significant and direct correlation existed between P4 and surfactant/co-surfactant ratio. The self-emulsifying drug delivery system formulations showed drug permeability through the rat intestine 2.76 times more, compared with the control.

**Conclusions:**

This study demonstrated that physicochemical properties, in vitro release and rat intestine permeability were dependent upon the contents of S/C, water and oil percentage in formulations.

## 1. Background

The oral route is one of the most preferred ways for chronic drug therapy; but the drug dissolution is usually a rate-determining step of the absorption processes for poorly water soluble drugs (1). Approximately 40% of marketing products are poorly soluble or lipophilic compound that lead to restricted oral bioavailability, high intra and inter subject variability and a possible increase in dose (2). To solve this problem, numerous methods such as solid dispersions, liposomes, use of cyclodextrins, nanoparticles, salt formation and etc. are utilized (3-5).

Lipid base formulation (LBF) is a useful method for enhancing oral bioavailability of class II drugs. Various types of LBF exist such as emulsion, self-emulsifying drug delivery systems (SEDDS), self-micro-emulsion drug delivery systems (SMEDDS), solutions or suspensions of the drug in lipid medium (6). SEDDS is one type of LBF that is defined as isotropic mixtures of natural or synthetic oils non-ionic surfactants or one or more hydrophilic solvent and co-solvents/surfactant. These formulations are having the droplet size in the range of 200 nm-5 µm and the dispersion has a turbid appearance (1). SEDDSs are stable preparations that increase the drug dissolution, provided by a large interfacial area of dispersion in oral administration. These systems form fine emulsions in the gastrointestinal (GI) tract with mild agitation, provided by gastric mobility and provide a large interfacial area for drug partitioning between oil and water phases, which increases in solubility and expand absorption (7). Potential advantages of these systems include the increased oral bioavailability, reduced in needed dose, controlled drug delivery, selective drug targeting (2), and advanced intestinal lymphatic transport of drugs that would be useful in reducing first pass of the drugs, such as carvedilol, and nimodipine (8). The higher surfactant level typically present in SEDDS formulations can lead to GI side-effects, and a new class of supersaturable formulations, including supersaturable SEDDS (S-SEDDS) formulations, have been designed and developed to decrease the surfactant side effects by using polymer as a precipitation inhibitor with a conventional SEDDS formulation (9).

Carvedilol is an arylethanolamine and a racemic mixture of two enantiomers that contains a nonselective β-adrenergic blocking agent with α 1-blocking activity that is used in the treatment of angina pectoris, mild to moderate hypertension, and chronic heart failure. Carvedilol poorly dissolves in water that limits drug absorption and delays onset time (10, 11). SEDDS is a strategy for increasing oral bioavailability and bioequivalence of poorly-water-soluble and lipophilic drugs. In our study Carvedilol SEDDS was evaluated to improve the dissolution rate, following by oral absorption of carvedilol.

## 2. Objective

The purpose of the present study was to prepare and develop a stable formulation for SEDDS to enhance the solubility, release rate, and oral absorption of the poorly-water-soluble drug, carvedilol.

## 3. Materials and Methods

### 3.1. Materials

Carvedilol was obtained from Dr. ABIDI’s Pharmaceutical laboratory. Labrafil M 1944CS, and Labrafac PG were gifts from the GATTEFOSSE Company (France). Oleic acid and Span 20 were obtained from Merck (Germany) Inc.; also, hydroxypropyl methylcellulose (HPMC) and Poloxamer were obtained from Sigma-Aldrich Corporation. Dialysis bag was purchased from the Toba Azema Co. Tehran, Iran.

### 3.2. Animals

A total of 24 adult male Wistar rats, four months of age, weighing 189 ± 12.1 g were purchased from the laboratory Animals Care and Breeding Center of Ahvaz Jundishapur University of Medical Sciences, Ahvaz, Iran. All rats were sacrificed using chloroform then animal intestines were removed and divided into four equal parts. All parts of intestine were washed in a cold Ringer’s solution. The experiment was performed in accordance with the guidelines for the use of animals in Ahvaz Jundishapur University of Medical Sciences. The guidelines used were prepared by the National Academy of Sciences and published by the National Institutes of Health.

### 3.3. Methods

#### 3.3.1. Construction of Ternary Phase Diagram

To obtain a concentration range of components for the existing boundary of SEDDS, pseudo-ternary phase diagrams were constructed using the water titration method (12). Two ternary phase diagrams were prepared with the 2:1 and 4:1 weight ratios of Labrafil M 1944CS/Labrafac PG. Oil phase (oleic acid) and the surfactant mixture were then mixed at the weight ratios of 1:9, 2:8, 3:7, 4:6, 5:5, 6:4, 7:3, 8:2, and 9:1 (7). These mixtures were diluted drop wise with double distilled water, under moderate agitation.

#### 3.3.2. Formulation of SEDDS

Various amounts comprising materials either of surfactant, co-surfactant, oil or with a constant amount of oil, HPMC and Poloxamer, were formulated by admixing the components (Table 1). Then carvedilol with a defined amount 1% of total formulation was added to the mixture, shaked well and then kept at 37°C for a time period necessary to solve the drug. We obtained the 2:1, and 4:1 weight ratios of Labrafil M 1944CS/Labrafac PG; thus, two series of formula was obtained٫ which in both surfactant and co-surfactant were Labrafil and Labrafac PG, respectively (13).

**Table 1.** Different Amount of Compounds in the SEDDS Formulations of Carvedilol ^a^

	Factorial Design Condition	O, g	S, g	CoS, g	O/s ratio	S/CoS Ratio	Polymer	Amount of Polymer	Drug, %	Amount of Drug
**1**	+ + +	12	4	1	3.1	4.1	Poloxamer	0.15	1	0.17
**2**	+ + -	12	4	1	3.1	4.1	HPMC	0.15	1	0.17
**3**	- + +	12	12	3	1.1	4.1	Poloxamer	0.15	1	0.27
**4**	+ - +	12	4	2	3.1	2.1	Poloxamer	0.15	1	0.18
**5**	- + -	12	12	3	1.1	4.1	HPMC	0.15	1	0.27
**6**	+ - -	12	4	2	3.1	2.1	HPMC	0.15	1	0.18
**7**	- - +	12	12	6	1.1	2.1	Poloxamer	0.15	1	0.3
**8**	- - -	12	12	6	1.1	2.1	HPMC	0.15	1	0.3

^a^ Abrevations: CoS, co-surfactant; O, oil; S, surfactant; O/S ratio, oil/surfactant; S/CoS ratio, surfactant/co-surfactant.

### 3.4. Visual Observation

For assessment of self-emulsification properties of formulations, 1 mL of each formulation was added in 0.1 N of hydrochloric acid (50 mL) under persistent stirring (60 rpm) at 37°C. Then spread ability tendency to emulsify and progress the emulsion droplets were observed. The formulations were classified as clear, non-clear, stable or unstable. Refractometric indexes of various formulations were measured and compared with the 0.1 N hydrochloric acid.

### 3.5. Solubility Study

The solubility of carvedilol in various oil, surfactant, co-surfactant was measured as follow: 5 mL of each selected vehicle (shown in Table 2) was added to excess amount of carvedilol and stirred for 30 minutes at 37°C, and then for one day (24 hours) at room temperature. Afterwards, the solution was centrifuged at 3000 rpm for 15 minutes and the supernatant was cleared, and the amount of dissolved drug was determined using a ultraviolet/visible (UV) spectrophotometer at proper wave length (14).

**Table 2.** The Solubility of Carvedilol in Various Oils (n = 3) ^a^

Oil	Solubility, mg/mL
**Oleic acid**	12.23 ± 0.56
**Labrafac PG**	0.46 ± 0.005
**Caster oil**	1.14 ± 0.17

^a^ Data are presented as Mean ± SD.

### 3.6. Carvedilol Assay

The amount of drug released and permeated through the rat intestine was determined using UV spectroscopy at a wavelength of 246 nm. The validity of assay method, including linearity repeatability accuracy and limit of quantification (LOQ) were calculated.

### 3.7. Droplet Size Analysis

After diluting formulations (1 mL) in 100 mL of 0.1 N hydrochloric acid solution, the particle size of formulations was determined and measured using particle size analyzer and Scatterscop 1 Qudix (12).

### 3.8. Drug Release Study From SEDDS

Franz diffusion cells (area 3.4618 cm^2^) with a cellulose membrane were used to determine the release rate of carvedilol from different SEDDS formulations. The cellulose (molecular weight 12000 G) membrane was first hydrated in the distilled water solution at 25°C for 24 hours. The membrane was then clamped between the donor and receptor chambers of the cells (14). Then diffusion cell was filled with 30 mL of 0.1 N hydrochloric acid. The receptor medium was constantly stirred by externally driven magnetic bars at 200 rpm throughout the examination. The blank SEDDS formulation without drug was used.

Carvedilol SEDDS formulations containing a defined amount of carvedilol (1 mL) was accurately weighted and placed in donor compartment. At 0.5, 1, 2, 3, 4, 5, 6, 7, 8 and 24 hour time intervals, 2 mL sample was removed from receptor for spectrophotometric analysis and replaced immediately with an equal volume of fresh 0.1 N hydrochloric acid (similar to gastric fluid). Samples were determined by UV visible spectrophotometer (BioWave II, WPA) at 246 nm.

The results were plotted as cumulative released drug percent versus time. Drug release from SEDDS formulations has been explained by fitting on kinetic models in which commonly used models such as zero order, first order, second order, 3/2 root of mass, linear and log wagner, Hixson-Crowell, Weibull, Korsmeyer-Peppas, Higuchi models, and the model with higher r^2^ had been selected (15).

### 3.9. Evaluation of Permeability of Drug from Rat Intestine

In order to assess the permeability, 1 mL of prepared formula was mixed with 1 mL 0.1 N hydrochloric acid and poured into the intestine and closed from both sides. Then, intestine was kept in 50 mL hydrochloric acid (0.1 N) for 4 hours at 37°C ± 0.5. The sampling was done at 0.5, 1, 2, 3, 4 hour time intervals followed by one hour intervals and absorption of the samples was determined by UV-visible spectrophotometer. The same test was performed for the saturated suspension of carvedilol and thus the amount of passed drugs between SEDDS and suspension were compared. Percentage of response to the drug permeated after four hours and the effects of independent variables on it were studied (13).

### 3.10. Statistical Analysis

In this research, the unpaired-two tailed t-test was used for statistical analysis of different formulations of the drug permeated through the intestine compared with the blank formula (without drug) and also to compare the effect of SEDDS and suspension on the amount of permeated drug. P < 0.05 was considered statistically significant. The Levene's test was used for homogeneity of variance. Also, ANOVA and multiple regressions were applied to simultaneously evaluate the relationship between several variables. Minitab 16 software was used for generating and evaluating the experimental design as well as evaluating the effect of variables on responses.

## 4. Results

### 4.1. Validity of Drug Measurement Method

The correlation coefficient for the concentration-absorbance was r^2^ = 0.998, which means that 99.8% of the absorbance values are estimated by the concentration. Regression analysis showed a significant relationship between concentration and light absorbance (P = 0.001). The lack-of-fit in this research was not significant (P = 0.167), which appears in the estimated absorbance changes. Accuracy of measurement showed those concentrations that were close to the actual values. Repeated surveys accountability in measurement methods within and between days for carvedilol displayed the desired repeatability of quantification method on different days and caused nearly-the- same operation as well as error-free results. All the concentrations observed in this research were higher than the LOQ (0.000365 mg/mL).

### 4.2. Solubility Studies

The strength of oil phase in drug solubility is an essential factor in the efficacy of SEDDS formulation. Solubility studies were performed to identify suitable oil that has the good solubilizing capacity for Carvedilol. Solubility in various oils is shown in Table 2. Among the used oils, the oleic acid and Labrafac PG showed respectively maximum and minimum solubility for carvedilol. Oleic acid (HLB = 4) had more strength to solve carvedilol than Labrafac PG (HLB = 2) Hence, it seems that in the present research, with increasing oil-phase of HLB, the drug’s solubility increased. Therefore, oleic acid was used as oil phase in SEDDS carvedilol formulations; moreover, Labrafil (HLB = 4) and Labrafac PG were used respectively as surfactant and co-surfactant. The saturated solubility for carvedilol in 0.1 N hydrochloric acid was 0.52 mg/mL ± 0.012 (n = 3).

### 4.3. Ternary Phase Diagram Study

**Figure 1.**
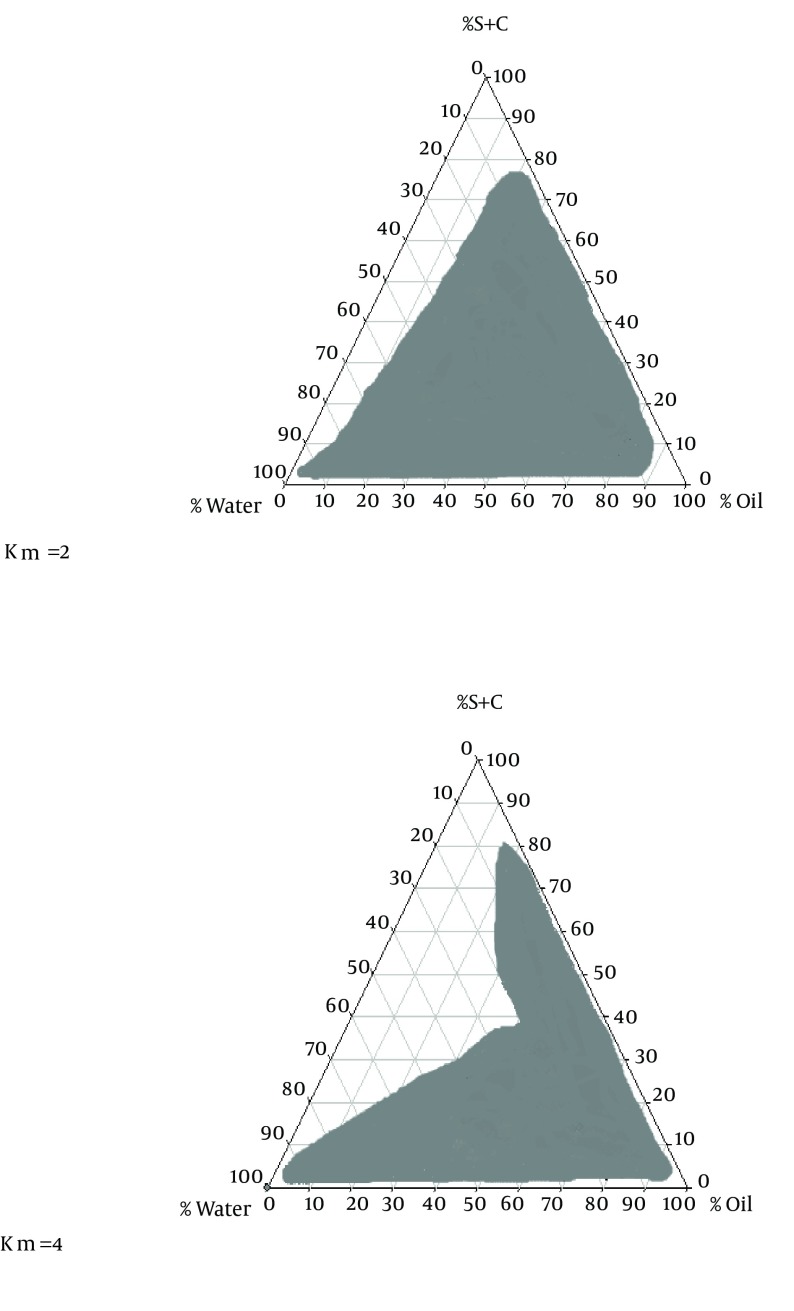
The Pseudo-Ternary Phase Diagrams of the Oil-Surfactant/Co-Surfactant Mixture–Water System at 2:1 and 4:1 Weight Ratio of Labrafil M 1944CS/Labrafac PG at Ambient Temperature Dark area represent emulsion region.

The phase diagram systems were composed of oil phase (oleic acid), surfactant (labrafil) and co-surfactant (labrafac PG). Oil, surfactant and co-surfactant were selected based on their drug solubility capacity, hydrophilic-lipophilic balance (HLB) values and ability of emulsion formation (15). Two phase diagrams were obtained at S/C of 2/1 and 4/1 are presented in Figure 1. The decrease in S/C ratio or surfactant/co-surfactant ratio (km = 2-4) cause an increase in numbers of points and area formation emulsion.

### 4.4. Characterization of the Carvedilol–Loaded SEDDS Preparations

#### 4.4.1. Particle Size Distribution

The SEDDS formulations had the mean particle size in the range of 0.248 to 0.910 µm. Multivariate regression was used for analyzing the correlation between independent variables and particle size of SEDDS formulations. The results show that the correlation between mean particle size was significant with polymer type (P = 0.003). The particle size of formulations was influenced by the type of polymer so that the mean particle size in SEDDS formulations prepared with HPMC (except formulation No. 8) had higher particle size compared to poloxamer formulations. Particle size and polydispersity index (PI) of the carvedilol SEDDS formulation are shown in Table 3.

**Table 3.** Polydispersity Index (PDI) and Particle Size of SEDDS Formulations Prepared by Poloxamer and HPMC (n = 3) ^a^

	Factorial Design Condition	Particle Size, µm	Polydispersity Index PDI
**1**	+ + +	0.299 ± 0.046	0.37 ± 0.009
**2**	+ + -	0.638 ± 0.045	0.38 ± 0.008
**3**	- + +	0.248 ± 0.059	0.38 ± 0.01
**4**	+ - +	0.363 ± 0.055	0.38 ± 0.03
**5**	- + -	0.91 ± 0.076	0.37 ± 0.03
**6**	+ - -	0.615 ± 0.143	0.41 ± 0.02
**7**	- - +	0.387 ± 0.231	0.38 ± 0.04
**8**	- - -	0.25 ± 0.0363	0.35 ± 0.009

^a^ Data are presented as Mean ± SD.

#### 4.4.2. Visual Observation Study

After addition of the various formulations to 0.1 N hydrochloric acid, the emulsion formation strength was evaluated using refractometric index (RI) and optical illusion method. After adding 0.1 N hydrochloric acid, a refractometric index for all formulations has difference with 0.1 N hydrochloric acid and water.

In the optical illusion method, formula obvious condition after 0.1 N hydrochloric acid from clear or non-clear, biphasic or an a phase after two hours post dilution with 0.1 N hydrochloric acid were studied (Table 4). Poloxamer formulations were translucent, and anaphase and HPMC formulations were milky and anaphase. Therefore, in poloxamer and HPMC formulations, the emulsion has been generated and percentages of used oil, surfactant and co-surfactant and type of polymer did not effect on emulsion formation. Because the effect of SEDDS formulations depends on their strength in the emulsion formation after entering the gastrointestinal tract, after the addition of the various formulations into 0.1 N hydrochloric acid, the emulsion formation strength was evaluated using both optical illusion method and refractivity index (RI) as the model of being transparent system. The closure of the formulations RI value to water and 0.1 N hydrochloric acid indicated the transparency property of the formulation being in the hydrochloric acid medium. 

In the optical illusion method, after adding 0.1 N hydrochloric acid from biphasic point, oil cells accumulation, and the amount of transparency, formula apparent condition immediately and 24 hours after dilution with 0.1 N hydrochloric acid were studied. The factor RI for all formulations prepared with HPMC and Poloxamer after adding 0.1 N hydrochloric acid did not have any significant difference with independent variables e.g. the phase behavior of SEDDS formulations and percentages of used oil, surfactant, co-surfactant and water were established stable emulsion, but in the area outside the range, stable emulsion has not been generated (Figure 1).

**Table 4.** The Emulsion Formation Strength Using Optical Illusion Method and Refractive Index (RI) of SEDDS Formulations (n = 3)^a^

	Factorial Design Condition	Refractive Index	Optical Illusion
**1**	+ + +	0.066667 ± 0.057735	translucent and anaphase
**2**	+ + -	0.033333 ± 0.057735	milky and anaphase
**3**	- + +	0.033333 ± 0.057735	translucent and anaphase
**4**	+ - +	0.066667 ± 0.115470	milky and anaphase
**5**	- + -	0.3 ± 0.1	translucent and anaphase
**6**	+ - -	0.1 ± 0.057735	milky and anaphase
**7**	- - +	0.166667 ± 0.057735	translucent and anaphase
**8**	- - -	0.066667 ± 0.057735	milky and anaphase

^a^ Data are presented as Mean ± SD.

### 4.5. In Vitro Drug Release

**Figure 2.**
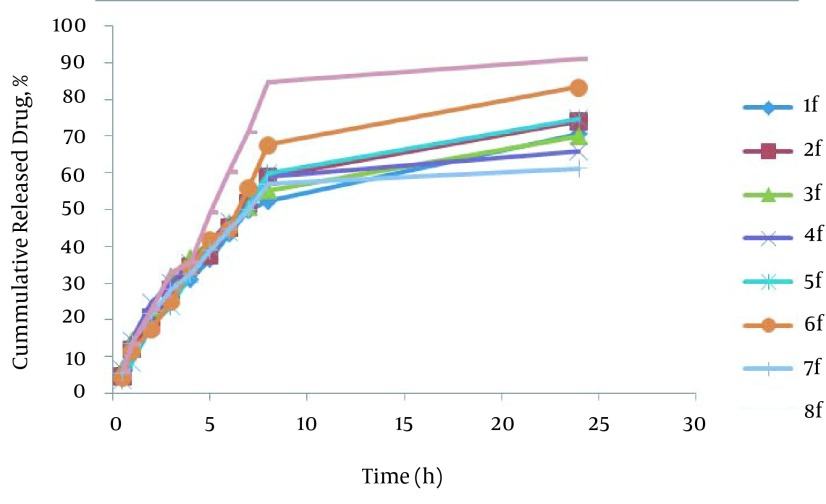
In Vitro Release Profile of SEDDS Formulation of Carvedilol

The percentage of drug released after 24 hours (R_24_) in the formulations prepared by poloxamer and HPMC were from 61.24-70.61 and 74.26-91.11 respectively. HPMC formulations have higher drug release (R_24_) than poloxamer formulations; also formulation No. 8 has maximum R_24_. In SEDDS carvedilol formulations, relationship between R_24_ with surfactant to co-surfactant ratio (S/C) (P = 0.817) and oil to surfactant ratio (O/S) (P = 0.190) was not significant and; however, type of polymer was significant (P = 0.001). It seems that no significant difference exists in particle size in their developed R_24_, because HPMC formulations have higher particle size than Poloxamer formulations. Figure 2 shows the release profiles of SEDDS carvedilol formulations. The percentage of drug released and kinetics of release in selected SEDDS formulations are presented in Table 5.

**Table 5.** Percent Release and kinetic Models Release of Selected SEDDS Formulation (n = 3)^a^

	Factorial Design Condition	Kinetic Model	R^2^	Intercept	Release, %
**1**	+ + +	Log wagner	0.9884	-1.1805	70.61 ± 1.03
**2**	+ + -	Log wagner	0.9872	-1.2335	74.26 ± 4.4
**3**	- + +	Log wagner	0.9917	-1.0793	70.12 ± 3.24
**4**	+ - +	Log wagner	0.9693	-1.0728	65.93 ± 5.16
**5**	- + -	Log wagner	0.9856	-1.3572	74.83 ± 2.23
**6**	+ - -	Weibul	0.9731	-1.3207	83.48 ± 0.84
**7**	- - +	Log wagner	0.9550	-1.1413	61.24 ± 2.91
**8**	- - -	Weibul	0.9562	-1.1607	91.11 ± 1.91

^a^ Data are presented as Mean ± SD.

### 4.6. Carvedilol Permeability From Rat Intestine

**Figure 3.**
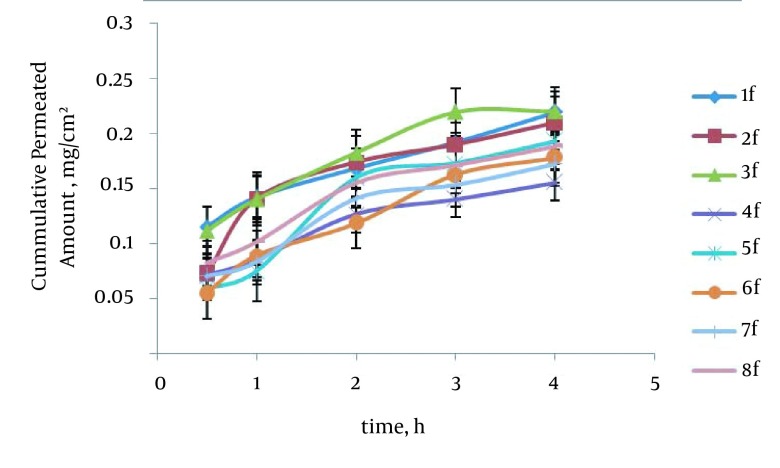
In Vitro Carvedilol Diffusion Through Rat Intestine From SEDDS Formulations

The maximum percentage of drug permeability after four hours (P_4_) was obtained 69.78% (formulation No. 3) in poloxamer formulations. The enhancement ratio in the formulation No. 3 was 2.76 times higher than those of saturated water solution of carvedilol. The relationship between surfactant to cosurfactant ratio (S/C) with P_4_ in the poloxamer and HPMC formulations was significant (P = 0.0017) indicating that in relation to the increased S/C ratio, the P_4_ has been increased. No significant difference existed between O/S ratio and type of polymer with P_4_ in the SEDDS formulations. Formulation No. 3 has the smallest particle size; therefore, it seems that the smallest size particulate has an essential role in the rat intestine permeability. Figure 3 represents in vitro carvedilol diffusion through the rat intestine from SEDDS formulations. The drug percent permeability through the rat intestine from various SEDDS formulations and control in different times are shown in Table 6.

**Table 6.** The Drug Percent Permeated Through Rat Intestine From Different SEDDS Formulations and Control ^a^

Time, h	Factorial Design Condition	The Drug Percent Permeated Through Rat Intestine				
		0.5	1	2	3	4
**1**	+ + +	35.55 ± 6.80	45.05 ± 6.65	53.23 ± 2.70	60.75 ± 5.42	69.68 ± 3.51
**2**	+ +-	22.90 ± 6.71	44.59 ± 15.59	55.16 ± 7.39	60.20 ± 7.46	66.44 ± 7.10
**3**	- + +	32.59 ± 4.23	41.13 ± 4.40	53.70 ± 5.33	59.46 ± 5.61	69.78 ± 5.52
**4**	+ - +	22.42 ± 6.78	26.98 ± 6.63	40.05 ± 7.26	44.50 ± 7.64	49.31 ± 8.57
**5**	- + -	18.60 ± 5.06	23.69 ± 3.90	50.52 ± 2.99	54.81 ± 5.22	61.29 ± 4.95
**6**	+ - -	17.25 ± 2.99	28.18 ± 2.59	37.50 ± 4.82	51.49 ± 4.83	56.40 ± 5.51
**7**	- - +	22.31 ± 4.50	26.27 ± 4.80	44.63 ± 2.70	48.63 ± 2.80	54.68 ± 4.34
**8**	- - -	26.01 ± 1.02	32.10 ± 1.34	49.18 ± 2.75	54.20 ± 2.29	59.70 ± 2.04
**Control**	-	7.36 ± 0.72	12.06 ± 0.53	16.23 ± 0.70	20.14 ± 0.56	25.33 ± 0.48

^a^Data are presented as Mean ± SD, n = 5

## 5. Discussion

There are numbers of potential mechanisms whereby SEDDS formulations may increase bioavailability; and especially in the case of Carvedilol solubility (7, 9, 10). SEDDS can produce fine oil in water emulsion after dilution in GI fluids with mild agitation provided by gastric mobility and provide a large interfacial area for drug partitioning between oil and water phases and increase in solubility rate and extent of absorption (1). This study demonstrated that physicochemical properties, drug released and permeation were dependent upon the type of polymer٫ ratio of O/S and S/C in formulations. In solubility studies of carvedilol, oleic acid and Labrafac PG showed respectively maximum and minimum solubility for carvedilol. Oleic acid (HLB = 4) had more strength to solve carvedilol than Labrafac PG (HLB = 2). Hence, it seems that in the present research with increasing oil phase HLB, the drug’s solubility increased. Phase diagrams indicated more width emulsion region with a decrease in S/C ratio.

In the optical illusion method, after the increased 0.1 N hydrochloric acid, formula were observed; Poloxamer formulations were translucent and anaphase, and HPMC formulations were milky and anaphase. Therefore, in the poloxamer and HPMC formulations, the emulsion has been generated and percentages of used oil, surfactant and co-surfactant, poloxamer and HPMC had not effect on the emulsion formation. The particle size of the SEDDS formulations was obtained between 0.248 to 0.910 µm. The particle size of formulations was influenced by the type of polymer so that the mean particle size in the SEDDS formulations prepared with HPMC (except formulation No. 8) has higher particle size compared to poloxamer formulations. The results show that correlation between mean particle size with polymer type was significant (P = 0.003).

The drug percent released after 24 hours (R_24_) in the formulations prepared by poloxamer and HPMC ranged from 61.24-70.61% and 74.26-91.11%, respectively. The HPMC formulations have higher drugs released (R_24_) than poloxamer formulations. Furthermore, formulation No. 8 has maximum R_24_. In the SEDDS carvedilol formulations correlation between R_24_ with surfactant to co-surfactant ratio (S/C) (P = 0.817), oil to surfactant (O/S) (P = 0.190) was not significant and also type of polymer was significant (P = 0.001). It seems that no significant difference was seen between particle size and their developed R_24_ (P > 0.05), because HPMC formulations have higher particle size than Poloxamer formulations. The kinetic model of drug release represents that the model provided by Weibul and log Wagner are the best model to estimate drug release in the HPMC and poloxamer formulations, respectively.

In rat intestine permeability studies, the maximum percentage of drug permeability after four hours (P_4_) was obtained 69.78% (formulation No. 3) in poloxamer formulations. The relationship between surfactant to co-surfactant ratio (S/C) with P_4_ in poloxamer and HPMC formulations were significant (P = 0.0017), which indicated that increased S/C ratio was correlated with increase of P_4._

No significant difference was seen between O/S ratio and type of polymer with P_4_ in the SEDDS formulations. Formulation No. 3 had the smallest particle size and therefore it seems that the smallest size particulate has the essential role in the rat intestine permeability.

## References

[1] Gursoy RN, Benita S (2004). Self-emulsifying drug delivery systems (SEDDS) for improved oral delivery of lipophilic drugs.. Biomed Pharmacother..

[2] Obitte NC, Rohan LC, Adeyeye CM, Parniak MA, Esimone CO (2013). The utility of self-emulsifying oil formulation to improve the poor solubility of the anti HIV drug CSIC.. AIDS Res Ther..

[3] Pawar SK, Vavia PR (2012). Rice germ oil as multifunctional excipient in preparation of self-microemulsifying drug delivery system (SMEDDS) of tacrolimus.. AAPS PharmSciTech..

[4] Shaji J, Lodha S (2008). Response Surface Methodology for the Optimization of Celecoxib Self-microemulsifying Drug delivery System.. Indian J Pharm Sci..

[5] Talegaonkar S, Azeem A, Ahmad FJ, Khar RK, Pathan SA, Khan ZI (2008). Microemulsions: a novel approach to enhanced drug delivery.. Recent Pat Drug Deliv Formul..

[6] Rahman MA, Harwansh R, Mirza MA, Hussain S, Hussain A (2011). Oral lipid based drug delivery system (LBDDS): formulation, characterization and application: a review.. Curr Drug Deliv..

[7] Kohli K, Chopra S, Dhar D, Arora S, Khar RK (2010). Self-emulsifying drug delivery systems: an approach to enhance oral bioavailability.. Drug Discov Today..

[8] Kale AA, Patravale VB (2008). Design and evaluation of self-emulsifying drug delivery systems (SEDDS) of nimodipine.. AAPS PharmSciTech..

[9] Gao P, Morozowich W (2006). Development of supersaturatable self-emulsifying drug delivery system formulations for improving the oral absorption of poorly soluble drugs.. Expert Opin Drug Deliv..

[10] Wei L, Sun P, Nie S, Pan W (2005). Preparation and evaluation of SEDDS and SMEDDS containing carvedilol.. Drug Dev Ind Pharm..

[11] Othman AA, Tenero DM, Boyle DA, Eddington ND, Fossler MJ (2007). Population pharmacokinetics of S(-)-carvedilol in healthy volunteers after administration of the immediate-release (IR) and the new controlled-release (CR) dosage forms of the racemate.. AAPS J..

[12] Salimi A, Sharif Makhmal Zadeh B, Moghimipour E (2013). Preparation and characterization of cyanocobalamin (vit B12) microemulsion properties and structure for topical and transdermal application.. Iran J Basic Med Sci..

[13] Liu L, Pang X, Zhang W, Wang S (2007). Formulation design and in vitro evaluation of silymarin-loaded self-micro emulsifying drug delivery systems.. Asian J Pharm Sci..

[14] Thakkar PJ, Madan P, Lin S (2014). Transdermal delivery of diclofenac using water-in-oil microemulsion: formulation and mechanistic approach of drug skin permeation.. Pharm Dev Technol..

[15] Patil P, Joshi P, Paradkar A (2004). Effect of formulation variables on preparation and evaluation of gelled self-emulsifying drug delivery system (SEDDS) of ketoprofen.. AAPS PharmSciTech..

